# Supplementary material for: Chemokine Receptors CCR6 and PD1 Blocking scFv E27 Enhances Anti-EGFR CAR-T Therapeutic Efficacy in a Preclinical Model of Human Non-Small Cell Lung Carcinoma
Source: Int J Mol Sci. 2023 Mar 12;24(6):5424. doi: 10.3390/ijms24065424 (PMC10056525; doi:10.3390/ijms24065424)
Supplement: Supplementary file 1 [file ijms-24-05424-s001.zip › ijms-2166924-supplementary.pdf]

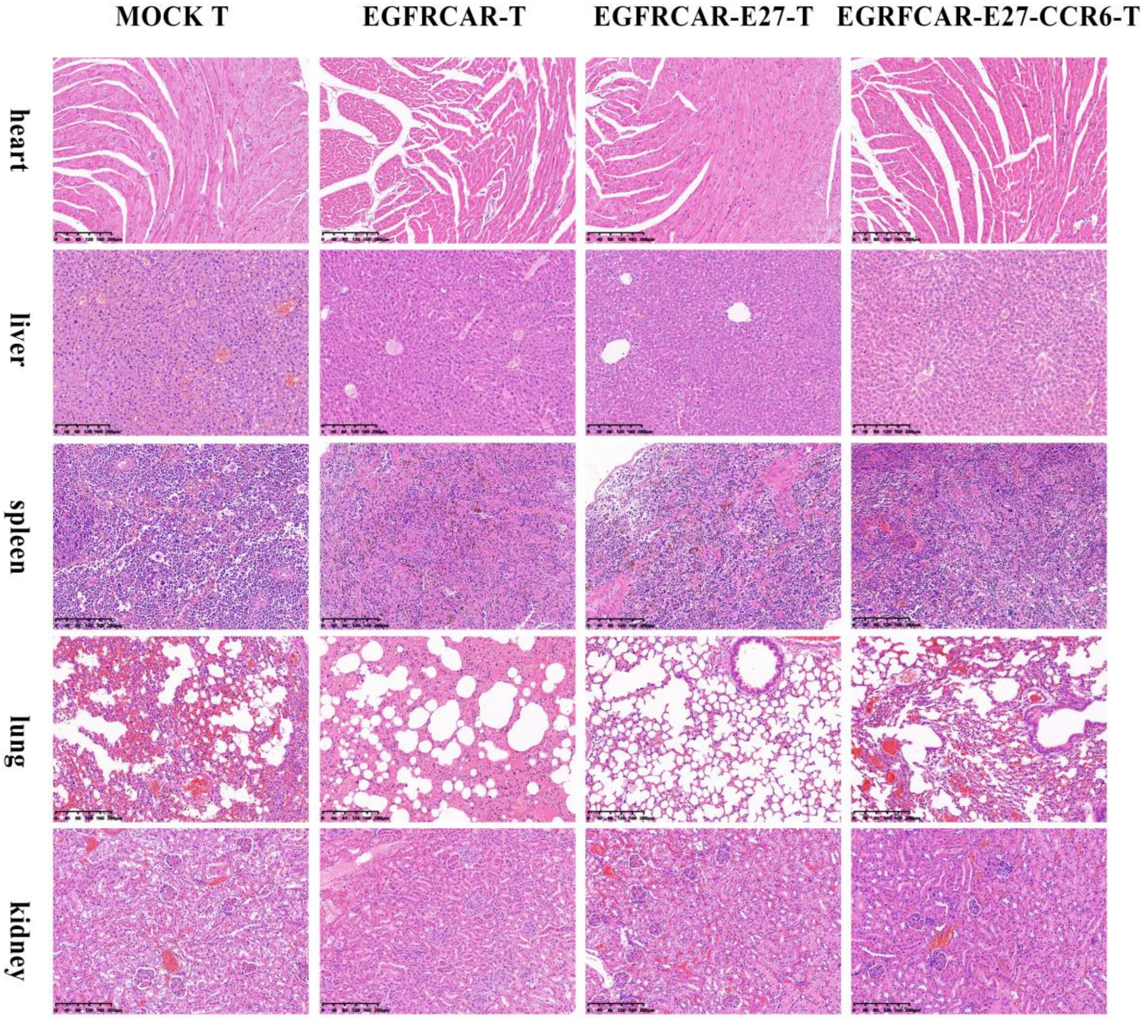

Figure S1. H&E staining was performed on major organs of mice, the safety of the CAR-T cells could be preliminarily evaluated based on the morphology and arrangement of the cells.
